# Antimicrobial resistance genes in the oral microbiome

**DOI:** 10.1038/s41432-025-01120-z

**Published:** 2025-03-06

**Authors:** Manas Dave, Rajpal Tattar

**Affiliations:** 1 The University of Manchester, Manchester, UK; 2 The University of Sheffield, Sheffield, UK

**Keywords:** Diseases, Dentistry

## Abstract

**A Commentary on:**

**Sukumar S, Rahmanyar Z, El Jurf H** **Q et al***.*

Mapping the oral resistome: a systematic review. *J Med Microbiol* 2024; 10.1099/jmm.0.001866.

**Design:**

This systematic review, without meta-analysis, aimed to map the oral resistome by analysing clinical studies that detected bacterial antimicrobial resistance genes (ARGs) in the oral cavity using molecular techniques.

**Data sources:**

The researchers used Medline, Embase, Web of Science, CINAHL and Scopus databases from January 2015 to August 2023.

**Study selection:**

This systematic review included cross-sectional or longitudinal clinical studies that detected ARGs using molecular techniques; specifically polymerase chain reaction (PCR) or next-generation sequencing (NGS) metagenomics for samples from the oral cavity (saliva, gingival biofilm, pulp, or oral mucosa). Studies were excluded if they were *in vitro* or animal studies, literature reviews and not focused on ARG detection.

**Data extraction and synthesis:**

Five reviewers independently screened titles and abstracts based on inclusion criteria. Full-text reports were then independently assessed for eligibility by three reviewers. Extracted data encompassed publication details, sample size, country, molecular methods used, number of ARGs detected, participants’ health status, antibiotic exposure, and sample location within the oral cavity.

**Results:**

Out of 580 initially identified studies, 15 met the inclusion criteria. These studies, published between 2015 and 2023 from 12 different countries, employed either PCR (n = 10) or NGS metagenomics (n = 5) to detect ARGs from a pool of 1486 participants (1 study did not report on the number of participants). PCR-based studies identified an average of 7 ARGs (range 1–20), while NGS studies identified an average of 34 ARGs (range 7–70). In total, 159 unique ARGs conferring resistance to 22 antibiotic classes were identified across six regions of the oral cavity. The supragingival biofilm and saliva exhibited the highest richness of ARGs, defined by the number of unique ARGs detected. Genes conferring resistance to 19 antibiotic classes were present in the supragingival biofilm. Notably, 49 ARGs, including tetracycline and macrolide resistance genes, were found across all sampled locations, indicating a widespread distribution within the oral cavity. Thirteen studies reported on bacterial species associated with ARGs. NGS studies identified a mean of 65 ARG-carrying bacterial species, compared to a mean of 4 species in PCR studies. Specifically, 25 ARG-carrying species were identified in PCR studies, while NGS studies identified 177 species. Four studies reported ARGs associated with streptococcal species implicated in distant-site infections such as infective endocarditis. ESKAPE pathogens (group of highly virulent multidrug-resistant bacteria) were detected with ARGs in various oral sites using both PCR and NGS methods. Comparisons between healthy and diseased states revealed that a healthy oral microbiome harbours a more diverse resistome at the antibiotic class level. The supragingival resistome demonstrated the richest composition in both health and disease, with tetracycline ARGs predominating in the supragingival and saliva resistomes in cases of dental caries.

**Conclusions:**

The analysis of the oral resistome from these 15 studies identified three ARGs present in all sites of the oral cavity, suggesting the presence of a core resistome. NGS studies provided greater insights compared to PCR studies; however, the overall research base is limited. Further comprehensive studies are necessary to fully map the oral resistome.

## GRADE Rating:



## Commentary

Antimicrobial resistance (AMR) represents a critical global health challenge, with the World Health Organisation identifying it as one of the top ten threats to humanity directly responsible for 1.27 million deaths in 2019^1^. The oral cavity, with its diverse and densely populated microbiome, is increasingly recognised as a reservoir for ARGs^2–4^. These genes can be transferred between bacterial species through horizontal gene transfer mechanisms such as conjugation, transformation, and transduction, potentially disseminating resistance to pathogenic bacteria both within and beyond the oral environment^4^.

The systematic review by Sukumar et al.^5^ provides a comprehensive analysis of the current state of knowledge regarding the oral resistome, the collection of all ARGs present in the oral microbiota. By focusing on studies employing molecular detection techniques such as PCR and NGS, the authors highlight the advancements in scientific ability to detect and characterise ARGs within the oral cavity.

The use of multiple databases and a broad time frame (from 2015 to 2023) ensured a wide capture of relevant studies. The inclusion criteria were well-defined, focusing on clinical studies that utilised molecular techniques to detect ARGs in human oral samples. The independent screening and assessment by multiple reviewers minimised selection bias and enhanced the reliability of the findings.

This systematic review demonstrates that NGS metagenomics identifies a higher number of ARGs and associated bacterial species compared to PCR. Specifically, NGS studies detected an average of 34 ARGs and 177 ARG-carrying species, whereas PCR studies identified an average of 7 ARGs and 25 species. This illustrates the superior sensitivity and comprehensiveness of NGS in characterising the oral resistome. The identification of ARGs across various regions of the oral cavity, with the supragingival biofilm and saliva showing the highest richness, provides valuable information on the distribution of resistance genes. The detection of ARGs conferring resistance to critical antibiotic classes, including tetracyclines and macrolides, across all sampled locations is particularly concerning given the clinical importance of these antibiotics.

The small number of included studies (n = 15) and the heterogeneity among them limit the generalisability of the findings. Variations were noted in study design, sample collection methods, participant demographics and analytical techniques. The predominance of PCR-based studies over NGS may reflect resource constraints, as NGS is more technologically demanding and costly. As NGS sequences all the DNA in a sample without a priori knowledge of the genes present, it inherently provides more comprehensive and unbiased insights into the resistome than PCR. NGS can detect a wider range of ARGs, including novel or unexpected genes, and can identify ARGs in a broader array of bacterial species. In contrast, PCR is restricted to a predefined set of ARGs and cannot detect genes outside of those targets.

There was no quality assessment of the included studies which limits the ability to fully appraise the validity and reliability of the findings. Moreover, the absence of a meta-analysis due to heterogeneity prevents drawing any definitive conclusions from pooled data.

The detection of ARGs in streptococcal species associated with infective endocarditis emphasises the importance of investigating oral bacteria that can cause systemic infections. Additionally, the identification of ESKAPE pathogens in the oral cavity is alarming, given their role in hospital-acquired infections and their multidrug-resistant nature.

Future research should prioritise the use of NGS metagenomics (over PCR) to provide a more comprehensive understanding of the oral resistome. Longitudinal studies examining the impact of factors such as antibiotic exposure, oral hygiene practices and systemic health conditions on the dynamics of ARGs in the oral microbiome are essential.

Practice points

- The research in this study does not change clinical practice. More research is needed in the oral microbiome and ARGS in oral microbiota and its impact on systemic disease and risk of developing antimicrobial-resistant infections.
- Prescription of antimicrobials should only be done according to guidelines and patients should be aware of the risk of antimicrobial-resistant infections.
